# Exchanges and Correspondents

**Published:** 1867

**Authors:** 


☞ EXCHANGES AND CORRESPONDENTS ☜ are respectfully requested to address the Editor of this Journal P. O. Box 1948, or at his office, 71 Dearborn Street. No other party whatever has any interest in, or control of, the JOURNAL, in any manner or form whatsoever. Any mistakes in direction of the JOURNAL, credits, &c., will be rectified at once, by addressing the Editor as above. His office has been recently removed to its present location, but the P. O. box remains as heretofore.

DR. J. ADAMS ALLEN,

*P. O. Box 1948, Chicago. Office of the* JOURNAL, *Room No. 2 McCormick Building, 71 Dearborn Street.*

